# Oral cancer incidence in Shanghai ---- a temporal trend analysis from 2003 to 2012

**DOI:** 10.1186/s12885-018-4582-4

**Published:** 2018-06-25

**Authors:** Jin-Ye Fu, Chun-Xiao Wu, Chen-Ping Zhang, Jing Gao, Jian-Feng Luo, Shu-Kun Shen, Ying Zheng, Zhi-Yuan Zhang

**Affiliations:** 1 Department of Oral & Maxillofacial - Head & Neck Oncology, Ninth People’s Hospital, College of Stomatology, School of Medicine, Shanghai Jiao Tong University, Shanghai Key Laboratory of Stomatology, No. 639, Zhi Zao Ju Road, Shanghai, 200011 China; 2 Department of Cancer Control & Prevention, Shanghai Municipal Center for Disease Control & Prevention, Shanghai, 200336 China; 3 Department of Epidemiology, Shanghai Cancer Institute, Shanghai, 200032 China; 4 Department of Biostatistics, School of Public Health, Fudan University, Shanghai, 200032 China

**Keywords:** Oral cancer, Epidemiology, Incidence, Temporal trend

## Abstract

**Background:**

Oral cancer is a serious problem owing to its poor prognosis and destruction of patients’ eating ability as well as facial appearance. Epidemiological studies can provide aetiological clues for prevention. The prevalence of oral cancer in densely populated cities in eastern China is unclear. The aim of the study is to analyse the incidence rates of oral cancer in Shanghai over the period 2003–2012 and estimate the temporal trends.

**Methods:**

Cases of oral cancer were retrieved from the Shanghai Cancer Registry system in the Shanghai Municipal Center for Disease Control & Prevention for the years 2003 to 2012. Information on the corresponding population was obtained from the Shanghai Municipal Bureau of Public Security. Age-standardised incidence rates were directly calculated according to the world standard population. An annual percent change model was employed to analyse the temporal trends of cancer incidence.

**Results:**

A total of 3860 oral cancer cases were reported, representing 0.69% of all malignancies in Shanghai during the 10-year study period. The mean age at diagnosis was 64 years. The age-standardised incidence rate was 1.34 per 100,000 person-years, with a male-to-female ratio of 1.41. Annually, the incidence rates increased by 3.83 and 2.54% for men and women, respectively. The increase was most noticeable in males aged 45–64 years.

**Conclusion:**

In Shanghai, the oral cancer incidence is relatively low. However, it is continuously increasing, especially among middle-aged males. This finding urges further investigations on the risk factors of oral cancer in this population, especially on changes in living patterns, such as the smoking, drinking, and dietary habits.

## Background

Oral cancer represents a serious problem worldwide. It is estimated that 442,000 cases were newly diagnosed during 2012 [1]. Globally, there are geographical variations in oral cancer incidence. The highest rate is reported in Melanesia of the Pacific region, with incidence rates of 22.9 per 100,000 for men and 16.0 per 100,000 for women [1]. Countries in South Asia, such as India, Pakistan, Sri Lanka, and Bangladesh, are also traditionally considered to have high occurrences of oral cancer. The aetiological factors behind the high incidences in these countries are mainly considered to include the usage of betel quid and various forms of tobacco [2]. Further, industrialised countries in central and eastern Europe are also experiencing high incidences of oral cancer, especially in the young population, indicating the presence of different aetiological risk factors [3].

In China, the incidence of oral cancer also widely varies among regions. High incidence areas are generally located in central and south China, where betel-nut chewing is popular. However, the prevalence in densely populated cities in eastern China remains unclear. In Shanghai, which currently has a residential population of over 14 million, a population-based cancer registration and reporting system has been established. The aim of the present study was to describe the incidence rates and temporal trends of oral cancer in Shanghai during the 10-year period of 2003 to 2012. The results will provide evidence-based clues for researches on the aetiology and prevention of oral cancer.

## Methods

Oral cancer incidence data between January 1, 2003 and December 31, 2012 were derived from the Shanghai Cancer Registry (SCR) system in Shanghai Municipal Center for Disease Control & Prevention, which is an associate member of the International Association of Cancer Registries. The SCR is a population-based cancer registry system that systematically collects, processes, and reports data on all newly diagnosed cancer cases among Shanghai residents. Doctors in hospitals throughout the city are required to report newly diagnosed cancer cases by using a standardised cancer reporting card to the SCR. Duplicates are consolidated in the data editing process. Death certification is used to help identify any cases missed in the routine reporting. The percent of cases identified via death certificate only (DCO) is 0.16% of the study.

The corresponding population denominators by age and sex were provided by the Shanghai Municipal Bureau of Public Security. The study was approved and the need for informed consent was waived by the institutional review board (IRB) of Shanghai Ninth People’s Hospital, Medical School of Jiao Tong University. There was no information to identify individual cases in the study.

The anatomical site locations for the included cases of oral cancer were the lip (10th edition of the International Classification of Diseases [ICD-10]: C00), mouth or oral cavity (C01–06) and oropharynx (C09–10), as detailed in Table 1. The epidemiological descriptions of oral cancer differ according to the anatomical subsites included in the definition. In some reports, oral cancer consists of cancers of the lip, tongue, mouth, salivary glands and all pharyngeal sites (C00-C14). As cancers of the oral cavity (C00-C06) and oropharynx (C09-C10) have similar pathological origins and clinical presentations, we grouped oral cavity and oropharyngeal cancers together as “oral cancer”, excluding cancers in the salivary glands (C07-C08) and other pharyngeal sites (C11-C14), in the present epidemiological study.

**Table 1:** International Classification of Diseases 10th edition (ICD-10) codes for oral cancer used for data collection in the study

ICD-10 code	Anatomical subsite
C00	Lip
C01	Base of tongue
C02	Other and unspecified parts of tongue
C03	Gingiva
C04	Floor of mouth
C05	Palate
C06	Other and unspecified parts of mouth
C09	Tonsil
C10	Oropharynx

The incidence rates are presented as cases per 100,000 population, and were age standardised by using the direct method to the world standard population, giving the age-standardised rates (ASRs). The significance of time trends in incidence was calculated by percent change (PC) and the annual percentage changes (APCs) with 95% confidence intervals [4]. Lifetime risk of developing oral cancer was calculated by using the cumulative approach. The significance level for comparisons and for APC trends was set at *P* < 0.05. Data management and all analyses were performed using SPSS 19.0 for windows (SPSS Inc., Chicago,IL).

## Results

Anonymous individual records by sex and 5-year-age groups were received from the SCR system, which has 100% coverage of the city’s population of approximately 14 million per year. A total of 3680 cases with oral cancer were reported during the study period: 2151 (58.5%) males and 1529 (41.5%) females, representing 0.69% of all malignancies in Shanghai from 2003 to 2012. As a result, the age-standardised incidence rate was 1.34/100,000 person-years (1.64/100,000 in males and 1.06/100,000 in females), with a male-to-female ratio of 1.41.

Table 2 shows the incidence rates for oral cancer by anatomical subsites during the 10-year study period: tongue cancer was the most common subsite for both sexes, followed by the oropharynx and gingiva in males, and gingiva and bucca in females.

**Table 2:** Incidence rates by anatomical subsites of oral cancer in Shanghai from 2003 to 2012

Anatomical subsite	Males			Females			Total		
	No. of cases	CR	ASR	No. of cases	CR	ASR	No. of cases	CR	ASR
Lip (C00)	94	0.14	0.07	107	0.16	0.07	201	0.15	0.07
Base of tongue (C01)	176	0.25	0.13	75	0.11	0.06	251	0.18	0.10
Tongue (C02)	586	0.85	0.46	553	0.80	0.38	1139	0.83	0.42
Gingiva (C03)	328	0.47	0.24	267	0.39	0.17	595	0.43	0.20
Floor of mouth (C04)	186	0.27	0.14	43	0.06	0.03	229	0.17	0.09
Palate (C05)	229	0.33	0.18	181	0.26	0.15	410	0.30	0.16
Bucca (C06)	207	0.30	0.16	215	0.31	0.13	422	0.31	0.14
Tonsil (C09)	135	0.20	0.10	63	0.09	0.05	198	0.14	0.07
Oropharynx (C10)	210	0.30	0.16	25	0.04	0.02	235	0.17	0.09
Total	2151	3.11	1.64	1529	2.22	1.06	3680	2.67	1.34

CR: crude incidence rate; ASR: age-standardised incidence rate

The incidence rates are reported as the number of cases per 100,000 person-years at risk

**Fig. 1:**
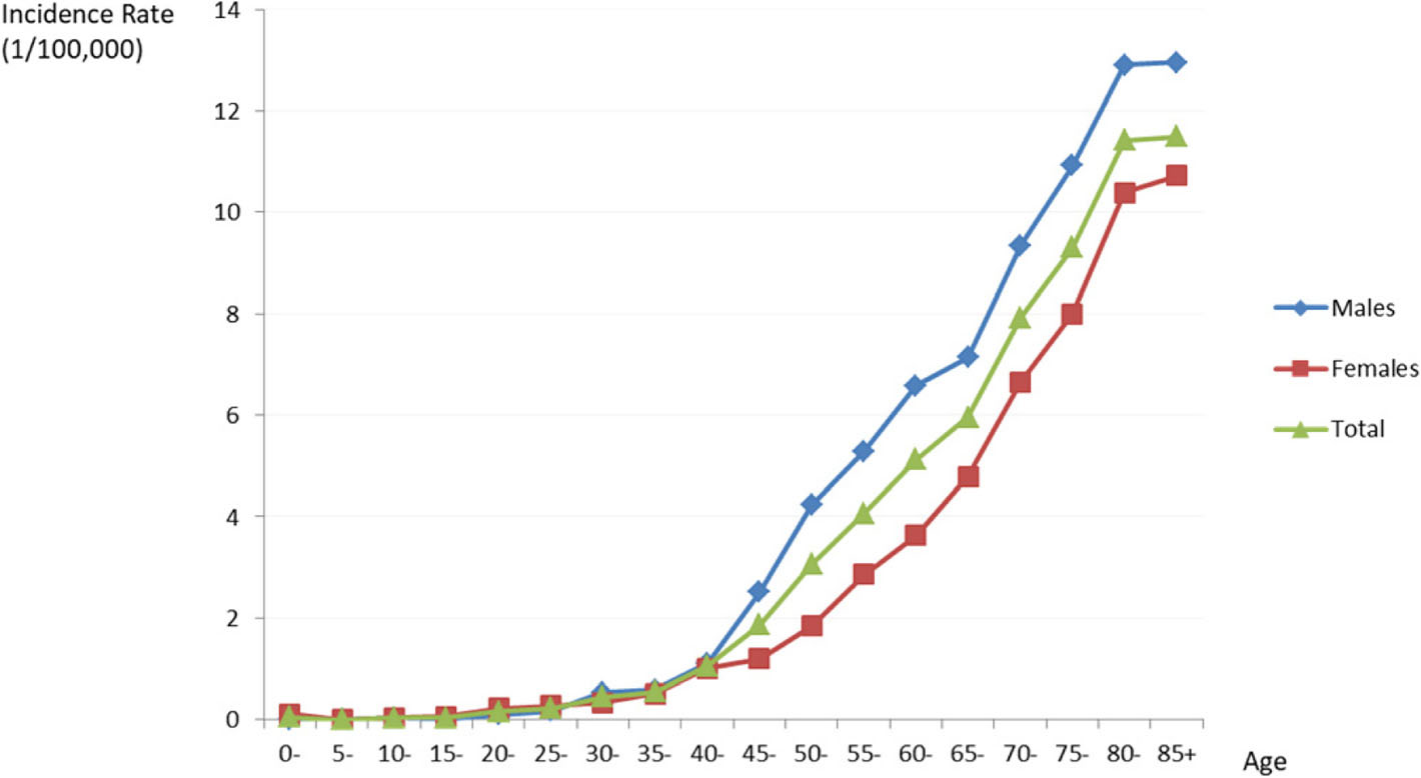
Age-specified incidence rates for oral cancer by sex in Shanghai from 2003 to 2012

During the 10-year period, the median age at the initial diagnosis of oral cancer in Shanghai was 64 years (males: 62 years; females: 69 years). Most cases were diagnosed after age 60, and 49.6% of cases were aged over 65 years. The lifetime risk of developing oral cancer in Shanghai was 0.31% in the whole population (0.37% in males; 0.26% in females). Figure 1 shows the incidence rates of oral cancer for the 10-year study period by 5-year age groups and sex. Oral cancer was rare among the young population aged < 45 years, with no major sex differences. However, among the older age groups, the incidence significantly increased with age, with oral cancer being nearly twice as common in males as in females.

**Fig. 2:**
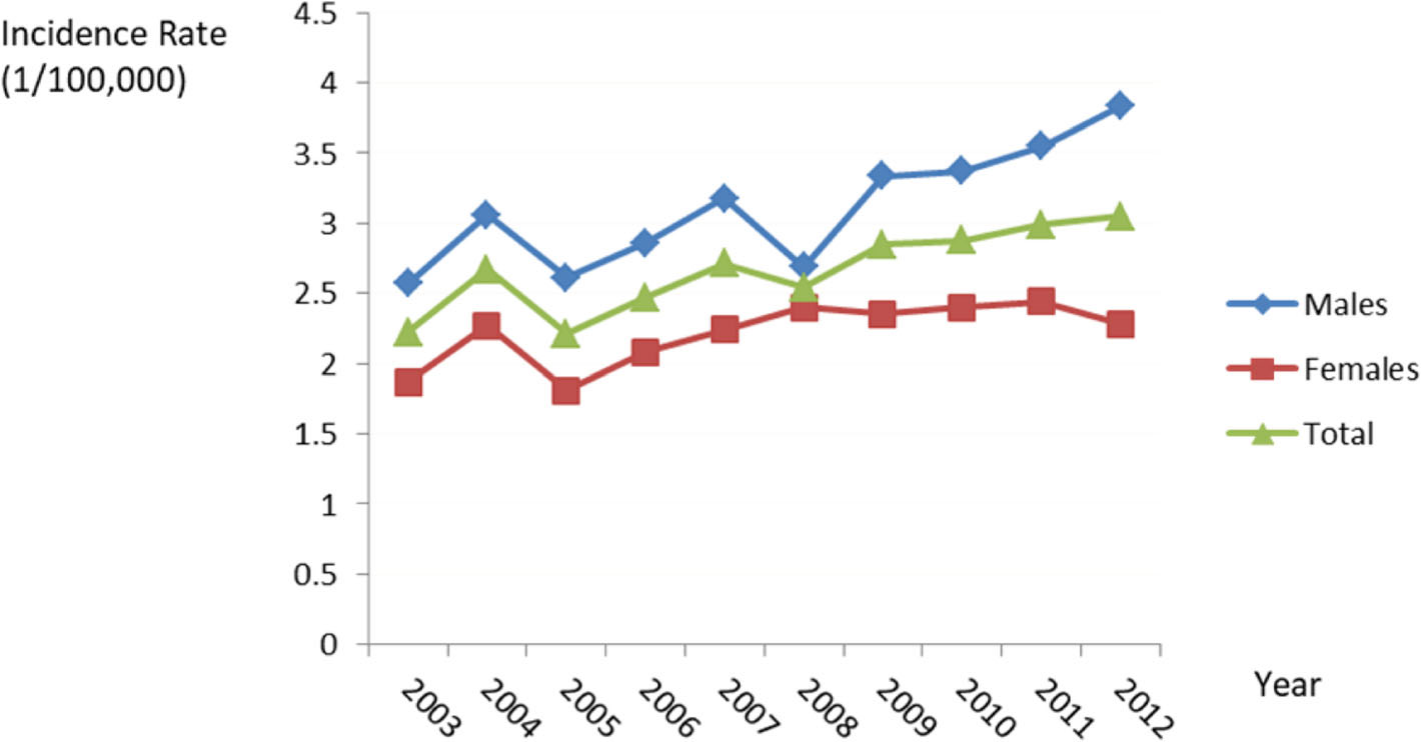
Annual incidence rates by sex for oral cancer in Shanghai from 2003 to 2012

Figure 2 highlights the yearly incidence trends by sex for all ages over time. In males, between 2003 and 2012, the crude rate for oral cancer in Shanghai rose from 2.57 to 3.83 per 100,000, with an annual percentage increase of 3.83 (*P* < 0.01). In females, the crude rate increased from 1.87 to 2.28 per 100,000, with an annual percentage increase of 2.54 (*P* = 0.02). However, the trends were not statistically significant in the ASR analysis (*P* > 0.05; Table 3).

**Table 3:** Yearly trends in the incidence of oral cancer in Shanghai from 2003 to 2012

Incidence rate	Sex	PC(%)	APC(%)	95% CI Lower	95% CI Upper	*t*-value	*P*-value
Crude rate	Males	31.11	3.83	1.74	5.95	4.27	< 0.01
	Females	14.00	2.54	0.52	4.6	2.91	0.02
	Total	23.61	3.29	1.67	4.93	4.73	< 0.01
ASR	Males	3.21	0.84	−1.63	3.38	0.78	0.46
	Females	−9.05	−0.12	−2.42	2.24	−0.12	0.91
	Total	−0.63	0.61	−1.43	2.69	0.68	0.52

ASR: age-standardised incidence rate; PC: percentage change; APC: annual percentage change; CI: confidence interval

Table 4 shows the incidence trends over time by age group (< 45 years [“younger”], 45–64 years [“middle-aged”], and ≥ 65 years [“older”]) for both males and females. The increasing trend was most noticeable in middle-aged males. In this group, the incidence rate increased by 52.99% between 2003 and 2012, with a significant APC of 6.26 (P < 0.01). However, in the other age groups, the trends were not statistically significant in the APC analysis.

**Table 4:** Comparison of temporal trends for oral cancer incidence by age and sex in Shanghai from 2003 to 2012

Year	Males(/100,000)			Females (/100,000)		
	< 45 y	45–64 y	≥65 y	< 45 y	45–64 y	≥65 y
2003	0.25	3.40	10.02	0.26	2.18	6.29
2004	0.47	4.05	11.19	0.26	2.40	8.32
2005	0.55	3.06	9.77	0.26	1.64	6.90
2006	0.31	3.62	10.84	0.36	2.15	7.09
2007	0.51	4.30	10.41	0.62	2.19	7.06
2008	0.26	4.00	7.92	0.55	1.88	8.75
2009	0.45	4.26	10.80	0.40	2.39	7.56
2010	0.27	5.11	8.79	0.28	2.66	7.39
2011	0.30	5.67	8.24	0.37	2.13	8.33
2012	0.33	5.72	9.80	0.25	2.34	7.26
PC(%)	−11.10	52.99	−14.96	18.29	−2.40	6.75
APC(%) [95% CI]	−2.25 [−9.62,5.72]	6.26 [3.43,9.17]	−1.89 [−4.61,0.9]	1.64 [−6.9,10.96]	1.46 [− 2,5.03]	1.25 [− 1.27,3.83]
*t*-value	−0.67	5.18	− 1.57	0.43	0.96	1.14
*P*-value	0.52	< 0.01	0.15	0.68	0.36	0.29

PC: percentage change; APC: annual percentage change; CI: confidence interval

## Discussion

Although the incidence of oral cancer in Shanghai is relatively low, it has been continuously increasing between 2003 and 2012. In the present study, 3680 cases of oral cancer were newly diagnosed during the 10-year period in Shanghai. The annual number increased from 297 in 2003 to 434 in 2012. Underlying this finding, our results indicate that the incidence rates increased significantly in middle-aged males.

An increase in the incidence of oral cancer has still been reported in some countries. In Europe, a rising incidence of oral cancer was first noted in Denmark in the middle of the twentieth century [5], and has since been consistently reported across the continent [6–9]. A recent epidemiological study showed a continuing increase in oral cancer incidence in the UK, with regional variation [10, 11]. North East of England had highest incidence, the South West and the East Midlands reported the most consistent increases, and the lowest incidence found in London. Scotland, where the rates are higher than in other parts of the UK, is experiencing a dramatic increase in younger age groups of both sexes [7]. Hungary, which is known to have a high incidence of oral cancer, is still experiencing increasing rates, which have nearly doubled in recent decades [12]. In some regions, the incidence trends of oral cancer differed between sexes. France once had the highest incidence rate of oral cavity and oropharyngeal cancer in Europe [13] and continued to increase until early 1980s [14]. In recent studies, the oral cancer strongly decreased in men, but strongly increased in women [3, 15]. The reason may be the changes of individual behaviours. Studies from the US have also suggested that the incidence is rising, with changes in the distributions of ethnicities and anatomic subsites reported [16, 17]. In eastern Asia, oral cancer has traditionally been uncommon; however, rising trends have recently been reported in the region [18–20].

In our analyses, the temporal trends of oral cancer incidence in Shanghai were significantly increased, as determined by the annual crude rates, in both sexes over the 10-year period. While calculated by the world standard population, the increase trend did not have statistical significance. It is due to the difference of the population structures between Shanghai population and the world standard. However, the crude rate reflects the real number of patients in a region and is essential for public health and prevention policies. In order to find out which age group had the most marked increase of oral cancer incidence, we classified all cases into young [< 45 years], middle aged[45–64 years], and the old [<=65 years].The increase was more marked in males aged 45–64 years than in other groups, though the cancer incidence remained the most common after 60 years, both for men and women.

The reasons for the above findings are speculative. Lifestyle habits may be one reasonable factor. Traditionally, excessive tobacco consumption and alcohol usage have been considered the major risk factors for oral cancer. They act both independently and synergistically on the development of oral cancer. Furthermore, other factors, including viral infection, especially the human papillomavirus (HPV), diet lacking in fruits or vegetables, regular consumption of hot and spicy meals, poor oral hygiene, and low socioeconomic status may also affect the aetiology of oral cancer. Thus, the recent changes in lifestyle in Shanghai need to be further investigated to better understand the rise of oral cancer incidence in this region.

Heavy smoking is still the main established risk factor for oral cancer incidence in most parts of the world. Historically, time trends of oral cancer incidence are found to be relevant to the temporal framework of smoking prevalence; cessation of smoking was associated with a sharply reduced risk of this cancer in one previous study. Taken together, these findings indicate a major role of smoking in the causation of oral cancer. A recent survey in the UK showed a continuing rise of oral cancer in regions with high consumption of tobacco products. In Scotland and northern England, where tobacco smoking is prevalent, the increase in the rates was more marked than in the rest of the UK, where adult smoking showed a modest decline. In the US, data from North Carolina, the leading location of tobacco production in the US, revealed a substantial oral cancer incidence in the state [21]. Conversely, an overall decrease in oral cavity cancer has been observed in the US, largely attributed to the success of public health programs aimed at reducing smoking [17]. Smoking habits are still common in Shanghai population. A survey investigated over 23,000 residents in Shanghai and showed that the smoking rate was 45.9% in men, with 45.6% male smokers consuming over 20 cigarettes per day. The percent of current smokers was similar to that reported 10 years before. [22].

Human papillomavirus infection has been identified as a rising cause of oropharyngeal cancer (OPC) incidence which significantly increased over the last 20 years in several countries. The England reported the incidence of potentially HPV-associated oral cancer increasing by 45.5% during the first decade of this century [11]. An increasing trend has also been observed for oropharyngeal cancers in the US, in contrast to the decreasing incidence of oral cavity cancer [17]. Studies in North American had detected HPV in up to 80% of oropharyngeal cancers, which indicated HPV being a major aetiologic factor [23]. Similar trends have been reported in Australia, Canada, and several European countries, mostly the economically developed countries [24]. Prophylactic HPV vaccination could be suggested for prevention. In our series, the number of OPC cases was small during the study period. The annual incidence rates were low that it was insufficient to analyze the trend for evaluating the potential impact of HPV in Shanghai. However, understanding the worldwide trends of HPV-associated oral cancer incidence could inform our public health for prevention policy.

Lip cancer is different from other sub-site oral cancers regarding the risk factors and the variation of incidence in a certain population. Sunlight exposure played a major role among fair-skinned people in lip cancer incidence. High incidence rates were reported in Oceania (account for 29% of all oral cancer) and some European countries [24]. With an increasing awareness of solar protection, the incidence rates became stable or decreasing [25]. Another risk factor for lip cancer, especially the lower lip cancer, is the habit of resting a cigarette on the lower lip while smoking. In global terms, eastern/southeast Asia had the lowest incidence of lip cancer. In our series, the lip cancer accounted for 5% of all oral cancers. The male to female ratio was 0.88. In the general global pattern, lip cancer occurred more often in men. However, a decrease in the male to female ratio for incidence has been observed in recent years [24]. The decreasing prevalence of smoking among men and slight increasing in women may have contributed [26]. But the explanation was not unambiguous. It needs further investigation to explore the reason for the higher incidence in females than in males in the report.

Alcohol consumption is traditionally major risk factor of oral cancer incidence. In western Europe, the steady increase of oral cancer in the past two decades has been suggested to result from increased consumption of alcohol [10, 27]. A 10-year study in Britain confirmed an increased likelihood of developing oral cancer among people who drank more than 20 units a week [28]. Alcohol drinking is also popular in the Chinese population. It has been reported that over 30% of Chinese males regularly consume alcohol [29]. The per capital alcohol consumption has been rising since the late 1980s, and is now even higher than in the recent decade [30]. In Shanghai, an analysis of alcohol drinking behaviours revealed that migrants had a higher drinking rate than residents [31]. As a fast-developing city, Shanghai attracts many young and middle-aged migrants.

Diet is another important constituent of lifestyle patterns. It has been reported that dietary habits play a role in the aetiology of oral cancer. Fruit, vegetables, and foods high in fibre are considered “healthier diets”, associated with reduced risks of oral cancer [32]. Geographical differences in food culture exist across China. There is a saying in China that “south sweet, north salty, east hot, and west sour”. Eating hot or spicy food for a long time is suspected to be related to the risk of oral cancer. It has been observed that hot meals and spicy food irritate and injure the oral mucosa, and increase keratinisation and the risk of viral infection, thus eventually leading to the development of cytological atypical changes and potentially oral cancer [33].

Shanghai is a coastal metropolis in eastern China. It is experiencing a rapid change in its population structure. A large number of people from all over the country have poured into the city, owing to its fast economic development. Most of them are young and middle-aged adults. As a result, the traditional living patterns of the native residents, including the eating, drinking, and smoking habits, are changing.

There are some potential limitations of this study. Most importantly, the study period of only 10 years was relatively short for a trend analysis. However, the SCR was not formally established until 2002. Prior to this, data on the cancer incidence in Shanghai were collected by another organisation and were confined only to the urban population. As the city is experiencing rapid changes, it is not rational to combine the data together for the analysis. It may well be that the trends will be more obvious if the study period is prolonged. Nevertheless, this study provides preliminary data on the prevalence of oral cancer in this region. This information may help promote aetiological investigations and the design of measures required for cancer prevention and control.

## Conclusion

This epidemiological study on oral cancer incidence in Shanghai showed an ASR of 1.34 per 100,000 person-years over the years 2003–2012. The male-to-female ratio was 1.41, and the median age at the time of oral cancer diagnosis was 64 years. Importantly, we found that the incidence rates for both men and women are increasing, with the increase being the most marked in middle-aged males. These findings may provide aetiological clues related to the incidence of oral cancer. In particular, the changing lifestyle habits of the population and their effects on the incidence of oral cancer need further investigation.

## Abbreviations

APC: Annual percentage change
ASR: Age-standardised rate
DCO: Death certificate only
HPV: Human papillomavirus
ICD-10: The 10th edition of the International Classification of Diseases
OPC: Oropharyngeal cancer
PC: Percent change
SCR: Shanghai Cancer Registry

## References

[1] Ferlay J, Soerjomataram I, Dikshit R (2015). Cancer incidence and mortality worldwide: sources, methods and major patterns in GLOBOCAN 2012. Int J Cancer.

[2] Chaturvedi AK, Anderson WF, Lortet-Tieulent J (2013). Worldwide trends in incidence rates for oral cavity and oropharyngeal cancers. J Clin Oncol.

[3] Ligier K, Belot A, Launoy G (2011). Descriptive epidemiology of upper aerodigestive tract cancers in France: incidence over 1980–2005 and projection to 2010. Oral Oncol.

[4] Bao PP, Zheng Y, Wu CX (2016). Cancer incidence in urban shanghai, 1973-2010: an updated trend and age-period-cohort effects. BMC Cancer.

[5] Møller H (1989). Changing incidence of cancer of the tongue, oral cavity, and pharynx in Denmark. J Oral Pathol Med.

[6] Karnov KSS, Grønhøj C, Jensen DH (2017). Increasing incidence and survival in oral cancer: a nationwide Danish study from 1980 to 2014. Acta Oncol.

[7] Robinson KL, Macfarlane GJ (2003). Oropharyngeal cancer incidence and mortality in Scotland: are rates still increasing?. Oral Oncol.

[8] van Dijk BA, Brands MT, Geurts SM (2016). Trends in oral cavity cancer incidence, mortality, survival and treatment in the Netherlands. Int J Cancer.

[9] Tarvainen L, Suuronen R, Lindqvist C (2004). Is the incidence of oral and pharyngeal cancer increasing in Finland? An epidemiological study of 17,383 cases in 1953-1999. Oral Dis.

[10] Conway DI, Stockton DL, Warnakulasuriya KA (2006). Incidence of oral and oropharyngeal cancer in United Kingdom (1990–1999) –– recent trends and regional variation. Oral Oncol.

[11] McCarthy CE, Field JK, Rajlawat BP (2015). Trends and regional variation in the incidence of head and neck cancers in England: 2002 to 2011. Int J Oncol.

[12] Warnakulasuriya S (2009). Global epidemiology of oral and oropharyngeal cancer. Oral Oncol.

[13] Ferlay J, Shin HR, Bray F (2010). Estimates of worldwide burden of cancer in 2008: GLOBOCAN 2008. Int J Cancer.

[14] Ménégoz F, Lesec’h JM, Rame JP (2002). Lip, oral cavity and pharynx cancers in France: incidence, mortality and trends (period 1975-1995). Bull Cancer.

[15] Ligier K, Belot A, Launoy G (2011). Epidemiology of oral cavity cancers in France. Rev Stomatol Chir Maxillofac.

[16] Shiboski CH, Shiboski SC, Silverman S (2000). Trends in oral cancer rates in the United States, 1973–1996. Community Dent Oral Epidemiol.

[17] Weatherspoon DJ, Chattopadhyay A, Boroumand S (2015). Oral cavity and oropharyngeal cancer incidence trends and disparities in the United States: 2000–2010. Cancer Epidemiol.

[18] Choi SW, Moon EK, Park JY (2014). Trends in the incidence of and survival rates for oral cavity cancer in the Korean population. Oral Dis.

[19] Zhang SK, Zheng R, Chen Q (2015). Oral cancer incidence and mortality in China. 2011 Chin J Cancer Res.

[20] Naganuma T, Kuriyama S, Kakizaki M (2008). Coffee consumption and the risk of oral, pharyngeal, and esophageal cancers in Japan: the Miyagi cohort study. Am J Epidemiol.

[21] Elter JR, Patton LL, Strauss RP (2005). Incidence rates and trends for oral and pharyngeal cancer in North Carolina: 1990–1999. Oral Oncol.

[22] Liu XX, Yao HH, Bao PP (2016). Smoking and secondhand smoke exposure among registered residents in shanghai. J Environ Occup Med.

[23] Cleveland JL, Junger ML, Saraiya M (2011). The connection between human papillomavirus and oropharyngeal squamous cell carcinomas in the United States. implications for dentistry J Am Dent Assoc.

[24] Shield KD, Ferlay J, Jemal A (2017). The global incidence of lip, oral cavity, and pharyngeal cancers by subsite in 2012. CA Cancer J Clin.

[25] Ariyawardana A, Johnson NW (2013). Trends of lip, oral cavity and oropharyngeal cancers in Australia 1982-2008: overall good news but with rising rates in the oropharynx. BMC Cancer.

[26] Moore S, Johnson N, Pierce A (1999). The epidemiology of lip cancer: a review of global incidence and aetiology. Oral Dis.

[27] Hindle I, Downer MC, Moles DR (2000). Is alcohol responsible for more intra-oral cancer?. Oral Oncol.

[28] Llewelyn J, Mitchell R (1994). Smoking, alcohol and oral cancer in south East Scotland: a 10-year experience. Br J Oral MaxillofacSurg.

[29] Xu XL, Zhao LY, Fang HY, et al. Status of alcohol drinking among population aged 15 and above in China in 2010—2012. J Hygiene Res 2016;45:534–537, 567 [in Chinese]. 29903318

[30] Su ZH, Hao W, Chen HX. Collaborate group for 2nd survey on alcohol drinking in five areas in China. Alcohol patterns, alcohol consumption and alcohol-related problems in five areas in China: Alcohol patterns and annual consumption. Chin Mental Health 2003;17:536–539[in Chinese].

[31] Zhou J, Ji DX, Pan ZG, et al. The analysis of alcohol drinking behaviors of migrant workers from rural to urban in shanghai, China. Fudan Univ J Med Sci 2015;42:18–22,30 [in Chinese].

[32] Filomeno M, Bosetti C, Garavello W (2014). The role of a Mediterranean diet on the risk of oral and pharyngeal cancer. Br J Cancer.

[33] Ahmed HG, Ebnoof SO, Hussein MO (2010). Oral epithelial atypical changes in apparently healthy oral mucosa exposed to smoking, alcohol, peppers and hot meals, using the AgNOR and Papanicolaou staining techniques. Diagn Cytopathol.

